# Infected Hematoma of the Femur

**Published:** 1918-12

**Authors:** 


**Infected Hematoma of the Femur.** By Dr. J. Bernard. *Bulletin de la Société de Chirurgie de Paris.* May 28, 1918.

A soldier presented among many other injuries a wound in the inferior third of the right thigh. The projectile was localized beneath the right " cul-de-sac " of the quadriceps, and removed.

After a fortnight, some slight edema appeared around the right knee and especially around the superior end of the tibia. An arthrotomy was practised but with no result. After a few days, the edema increased. Temperature rose to 39.5° C. The patient's general state was alarming.

Dr. Bernard diagnosed a lesion of the cancellous tissues of the femur, having determined secondarily arthritis and peri-arthritis.

A wide, U-shaped arthrotomy was practised, a resection was attempted; the synovial membrane was found to be quite red, swollen, and thickened with pus; the cartilages, the periosteum, the femoral and tibial epiphysis, and the condyles were injured and infected. The thigh was amputated above the inferior third of its length. Temperature fell rapidly, and the patient recovered. In that case, a very small lesion of the bone above the condyles was followed by very serious secondary infection of the marrow and cancellous tissues.

